# The Influence of Matrix Size on Statistical Properties of Co-Occurrence and Limiting Similarity Null Models

**DOI:** 10.1371/journal.pone.0151146

**Published:** 2016-03-04

**Authors:** Thomas Michael Lavender, Brandon S. Schamp, Eric G. Lamb

**Affiliations:** 1 Dept. of Plant Sciences, University of Saskatchewan, Saskatoon, Saskatchewan, Canada; 2 Dept. of Biology, Algoma University, Sault Ste. Marie, Ontario, Canada; University of Fribourg, SWITZERLAND

## Abstract

Null models exploring species co-occurrence and trait-based limiting similarity are increasingly used to explore the influence of competition on community assembly; however, assessments of common models have not thoroughly explored the influence of variation in matrix size on error rates, in spite of the fact that studies have explored community matrices that vary considerably in size. To determine how smaller matrices, which are of greatest concern, perform statistically, we generated biologically realistic presence-absence matrices ranging in size from 3–50 species and sites, as well as associated trait matrices. We examined co-occurrence tests using the C-Score statistic and independent swap algorithm. For trait-based limiting similarity null models, we used the mean nearest neighbour trait distance (NN) and the standard deviation of nearest neighbour distances (SDNN) as test statistics, and considered two common randomization algorithms: abundance independent trait shuffling (AITS), and abundance weighted trait shuffling (AWTS). Matrices as small as three × three resulted in acceptable type I error rates (p < 0.05) for both the co-occurrence and trait-based limiting similarity null models when exclusive p-values were used. The commonly used inclusive p-value (≤ or ≥, as opposed to exclusive p-values; < or >) was associated with increased type I error rates, particularly for matrices with fewer than eight species. Type I error rates increased for limiting similarity tests using the AWTS randomization scheme when community matrices contained more than 35 sites; a similar randomization used in null models of phylogenetic dispersion has previously been viewed as robust. Notwithstanding other potential deficiencies related to the use of small matrices to represent communities, the application of both classes of null model should be restricted to matrices with 10 or more species to avoid the possibility of type II errors. Additionally, researchers should restrict the use of the AWTS randomization to matrices with fewer than 35 sites to avoid type I errors when testing for trait-based limiting similarity. The AITS randomization scheme performed better in terms of type I error rates, and therefore may be more appropriate when considering systems for which traits are not clustered by abundance.

## Introduction

A tenet of community ecology is that there exists a set of rules that constrain community assembly [1] and that these rules are the combined result of species interactions and abiotic factors (i.e., environmental filtering)[2–4]. In an effort to determine these community assembly rules, a number of null models have been developed and have come into common use [5]. Null models testing for negative co-occurrence and limiting similarity are regularly used to evaluate the influence of competition on community structure [6–21]. While both models focus on linking community patterns to the competitive process, they do so in different ways. Co-occurrence null models test for patterns of segregation among species that may be the result of competitive exclusion, although it is clear that other processes may influence these patterns [22,23]. Limiting similarity null models (i.e. trait-based null models) test whether species found together in samples are convergent or divergent with respect to important functional traits; convergence is often taken as evidence for environmental (abiotic) filtering and divergence as evidence for biotic filtering (i.e., competitive filtering) as predicted under limiting similarity [24], although interpretations of patterns of convergence vary (e.g., [8,20]).

Both classes of null model have undergone some testing to determine whether they perform with acceptable type I and type II error rates [25–27]; however, this testing has been confined to a limited set of matrix sizes. For co-occurrence null models, Fayle & Manica [27] explored matrices ranging from 10 × 10 up to 100 × 100; however, they relied on the sequential swap algorithm, which is vulnerable to serial correlation [28]. Furthermore, both Gotelli [25] and Fayle & Manica [27] explored type II error rates using only highly structured, biologically unrealistic test matrices. Gotelli [25] explored a single biologically realistic matrix (17 × 19), but only for type I error rates. As such, understanding error rates for co-occurrence tests on small, biologically realistic matrices (specifically for type II error rates) requires further work. For limiting similarity null models, no comparable analysis of error rates has been carried out; however, studies have assessed the performance of related null models of phylogenetic dispersion [26,29]. It is clear from the phylogenetic analyses that performance is dependent on the combination of metric and null model [26,29]. Consequently, error rates for trait-based limiting similarity models require further attention. Finally, it is not clear how trait-based null model performance is affected by community matrix dimension.

There are a number of reasons why it is important to understand how these null models perform at the lower end of matrix dimensionality. Reduction of matrix size through small numbers of species or sites can adversely affect these null models by reducing both the number of ways that the matrix can be shuffled and the granularity of the C-Score values (C-Score is the mean number of 2 × 2 checkerboard sub-matrices {{1,0},{0,1}} per species pair in a community [30]). For example, a 3 × 3 matrix can be shuffled a maximum of three ways using the independent swap algorithm and has a granule size of 1/3. Granule size is the amount of increase or decrease that a single change in checkerboardedness within the matrix imparts on the C-Score (i.e., the minimum incremental change in C-Score); it is equal to one divided by the number of pairwise comparisons 1/(*r*(*r*– 1)/2) where *r* is the number of rows in the matrix. Granularity combined with the number of sites and matrix density (the number of non-zero cells in the matrix) determines the range of possible C-Score values. It is unknown if constraints on this range influences the error rates of the null models although Ulrich and Gotelli [31] have investigated one component of granularity, matrix fill, on null model performance.

The type I error rates of co-occurrence null models on mid- to large-sized matrices is reasonably well established [25,27,28]; however, several studies have used co-occurrence null models on matrices smaller than those for which error rates have been estimated (e.g., [31–38]). The conclusions of these studies rely on the stability of error rates at low matrix dimensionality.

For trait-based limiting similarity null models, no analysis of statistical performance has been carried out and nothing is known about the impact of variation in matrix size on their performance. Importantly, a broad range of community dimensions have been tested for patterns of limiting similarity using these null models (e.g., [39] species = 1,083, [40] species = 499, [41] species = 11 & 14); the proliferation of trait-based investigations has outpaced our efforts to understand their efficacy. As such, our understanding of the general trends across limiting similarity studies [5] may be impeded by problems related to error rates for particular focal matrix sizes. Error rate-related problems may explain why some studies have found support for limiting similarity (e.g., [17,24,39,42]) while many more have not (e.g., [13,20,37,41,43–47]) and why a recent review by Götzenberger et al. [5] found little support for limiting similarity.

As the number of studies using co-occurrence and limiting similarity null models increases, it remains uncertain as to the suitability of these tests across a broad range of matrix dimensions. In this study, we address this uncertainty by determining the statistical performance (i.e., type I and type II error rates) of commonly used co-occurrence and trait-based limiting similarity null models across a broad range of community dimensions.

## Materials and Methods

### Generating presence-absence matrices

We generated presence-absence matrices using a modified version of a method used by Ulrich and Gotelli [31]. Ulrich and Gotelli's original method was initially developed to produce ecologically realistic species abundance matrices. The process of generating presence-absence matrices consisted of creating synthetic matrices of *m* rows (species) by *n* columns (sites) with the number of sites each species occurs in determined by random sampling from a log-normal distribution (constrained between 0 and n). Species occurrences were randomly distributed across sites until its occurrences matched the total number of occupied sites. This was done for each species (row/species) in the matrix. Species incidences of zero were discarded in order to prevent the creation of degenerate matrices (matrices with empty rows; [25]) and each column of the matrix was checked to ensure that it contained at least one species incidence. In the event that a site did not contain any species, a row of the matrix was selected at random and the species occurrences shuffled among sites. This was repeated until no sites were empty.

### Trait generation

For the limiting similarity null model tests, trait values were generated by randomly drawing from a uniform distribution constrained to the set of numbers {*x* ∈ ℝ | 0 < *x* ≤ 100} and limited to two decimal places by truncation. Other schemes for generating trait distributions (e.g., [29,48,49]) are also possible. Given that there was no clear precedent for selecting one distribution over another, the uniform distribution seemed to be the best choice. The uniform distribution inherently lacks underlying patterns that could lead to clustered trait values and therefore influence error rates. Additionally, our goal was to examine a broad class of trait and presence-absence matrices without making assumptions concerning the underlying processes governing these trait distributions.

### Null models

For co-occurrence analyses, we used the fixed-fixed independent swap algorithm [25,50] in combination with the C-Score [30]. We used 30,000 swaps per randomisation as recommended by Lehsten et al. [51] and null distributions were generated from 5000 randomisations of the focal matrix. This combination of algorithm and metric is commonly used and is recognized as being among the best in terms of statistical performance (although see above for limitations of these analyses) [25].

For limiting similarity tests, we explored two null model randomization methods (below) in combination with two metrics: mean nearest neighbour trait distances (NN) and, standard deviation of nearest neighbour distances (SDNN). These test statistics were calculated for the community as means across site-level values (e.g., [20,42]). It is also possible to examine site-level patterns (e.g., [17,52]); however, the current approach benefits from treating sites as sample replicates, giving a better assessment of overall community-level patterns. The two randomization procedures examined were abundance-independent trait shuffling (AITS) and abundance-weighted trait shuffling (AWTS) [47]. AITS has been commonly employed (e.g., [11,13,17,47]) and consists of shuffling trait values among species without constraint. AWTS is an alternative method of shuffling trait values between species that preserves trait-abundance relationships [14,17,26,42,47]. Both randomization schemes are commonly used and neither has been shown to be a better choice statistically. While other null models exist, we use common approaches here to enable a very detailed analysis; any matrix-dimension effects observed are likely to apply broadly to several combinations of randomization scheme and test statistic.

### Type I error rate estimations

We estimated type I error rates for all matrices by evaluating each using the appropriate null model and test statistic(s). Because matrices were generated randomly, null models should fail to find significant patterns at least 95% of the time (p < 0.05). To test the effect of matrix dimension on type I error rates we generated 10,000 matrices for each combination of *m* species, *m* = {3, 4, 5,…, 17, 18, 19, 20, 25, 30, 35, 50}, by *n* sites, *n* = {3, 4, 5,…, 12, 13, 14, 15, 20, 25, 30, 35, 50, 75, 100, 150} for a total of 4,620,000 matrices.

For each matrix we calculated “observed” values for each test statistic (C-Score, NN and SDNN) as well as a distribution of “expected” values to determine the cumulative frequency of expected observations that were <, =, and > the observed value. We used three methods to assess significance. First, the norm for these types of analyses is to assess significance based on the number of expected values that are equal to or more extreme than the observed value (≤ or ≥), an inclusive p-value. Alternatively, we assess significance based on the number of expected values that are more extreme than the observed, without the equivalence criterion (< or >). The use of an exclusive p-value is consistent with normal hypothesis testing but is a more conservative test of significance versus an inclusive p-value (see [53] for a discussion of statistical significance). The exclusive p-value is relevant when the null distribution contains repeated values, as may be common for null models assessing small presence-absence matrices with small grain size. The third method is based on the standardized effect size (SES; see [54]) which has primarily been used to compare patterns across treatments (e.g., [36,55]). SES is less commonly used as a measure of significance [56]; for our purposes, |SES| > 1.96 was considered significant. Correspondence between significance as assessed by SES and p-values will vary with the normality of the null distribution. SES was calculated as (*Obs*–*Mean*)/*Std*, where *Obs* is the observed value for each test statistic, and *Mean* and *Std* are the mean and standard deviation respectively of the test statistics for randomized matrices. For small matrices it was common to have a standard deviation that was either zero or approaching zero, which resulted in extreme SES values (|SES| ≥ 20). These extreme values were associated with matrices that, when shuffled, resulted in extremely small or zero difference in the values for the test statistic (small grained matrices) and occurred with both the AWTS and AITS null models but were more prevalent with the AWTS-SDNN combination (proportion of outliers by method; AWTS-SDNN: 0.0592, AWTS-NN: 0.0010, AITS-SDNN: 0.0368, AITS-NN: 0.0005). These outliers were removed from the comparison of SES values but were retained for other analyses (see S1 Fig).

### Type II error rate estimations

Type II error rates represent the probability that real patterns of co-occurrence or limiting similarity go undetected by null models. A similar approach to the type I error rate estimations was used; however, for these tests, we greatly amplified the amount of pattern in each matrix and then tested the ability of null models to find that pattern. To do this both presence-absence matrices and trait values were randomly generated using the methods described above. The number of species and sites used for the co-occurrence tests were *m* species, *m* = {5, 10, 15, 20, 25, 30, 35}, by *n* sites, *n* = {3, 4, 5, …, 13, 14, 15, 20, 25, 30, 35, 50}. The number of species and sites used for limiting similarity tests were *m* species, *m* = {5, 10, 15, 20, 25, 30, 35}, by *n* sites, *n* = {3, 4, 5, …, 13, 14, 15, 20, 25, 30, 35, 50, 75, 100, 150, 200}. The co-occurrence null model used a reduced set of sites (max. *n* = 50) compared to the limiting similarity null models due to the substantially longer computational time required for those tests. For both co-occurrence and limiting similarity tests 10,000 presence-absence matrices were generated for each combination of *m* × *n*. This produced 1,260,000 matrices for co-occurrence tests and 1,540,000 matrices for limiting similarity tests.

To produce matrices with maximal or near-maximal C-Scores (i.e., with increased signal), the observed C-Score was determined for the generated matrix, after which each species was re-assigned among sites using a Fisher-Yates shuffle, which maintains row but not column totals [57]. In a minority of cases, this resulted in sites with no species and therefore altered matrix sizes. Some additional analyses confirmed that the Fisher-Yates shuffle did not alter our matrices in a way that affected our results (see Appendix A for details). Once all species were shuffled among sites, the C-Score was re-calculated. If the new C-Score was greater than the previous, the new matrix was stored. Each matrix was shuffled 10,000 times to ensure a maximal or near-maximal C-Score.

We used a similar process to maximize trait structure for the limiting similarity null models. Because one of the expectations under limiting similarity is that coexisting (i.e. positively co-occurring) species will differ with respect to relevant traits, we only added trait structure to matrices without significant negative co-occurrence. We used a pairwise test of species co-occurrence to rapidly select matrices with no negatively co-occurring pairs of species [58]; this approach does not require a null model test and sped up the process of adding signal. Trait values were generated using the same method as for the type I error rate assessments. Limiting similarity signal was maximized (or nearly so) by iteratively shuffling traits among species, and recalculating the test statistics. If the shuffled traits increased the test statistic (decreased for SDNN), it was stored and the process was continued. The trait distribution that maximized the observed limiting similarity after 100,000 iterations of this process was then tested using the null models. We estimated type II error rates as the proportion of matrices that null model tests indicated as having significant patterns of limiting similarity. This method of estimating type II error rates is consistent with previous methods (e.g., [25,26,29,31,59]). Because we were interested in trait divergence (NN) and evenness of trait spread (SDNN) as signals of limiting similarity, and because we maximized signal in these directions only, we calculated one-tailed p-values for our tests.

### Software

All analyses were written in Scala (Version 2.9.2) [Computer Language], available from http://www.scala-lang.org/downloads using IntelliJ IDEA Community Edition (Version 12.1.3) [Computer program], retrieved from http://www.jetbrains.com/idea/download/index.html and run on the Java VM (Version 1.6) [Computer software], available from http://www.oracle.com/technetwork/java/javasebusiness/downloads/java-archive-downloads-javase6-419409.html. Statistical analyses (non-null model) were carried out using the R Project for Statistical Computing (Version 3.0.3) [Computer software], available at http://cran.r-project.org. Plots were generated using the Lattice package [60]

## Results

### Type I Error Rate Estimation

**Fig 1:**
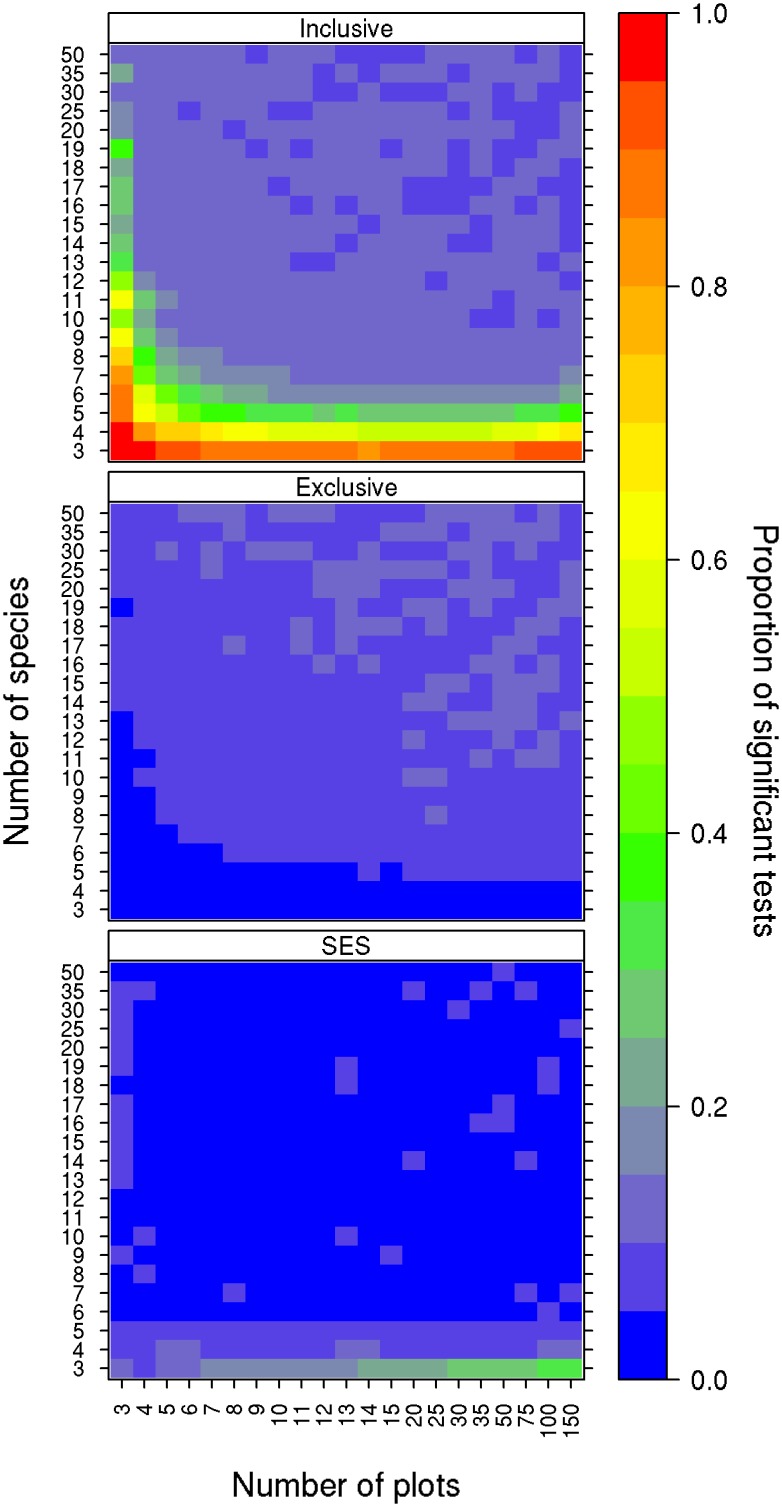
Type I error rates of the co-occurrence null model test. Each panel represents a different criterion for determining the significance of the null model: inclusive p-values (≤ or ≥), exclusive p-values (< or >), and SES. The colour of each cell indicates the proportion of the 10,000 null models that were significant for that combination of species by sites. Blue cells indicate lower type I error rates and red cells indicate higher type I error rates.

**Fig 2:**
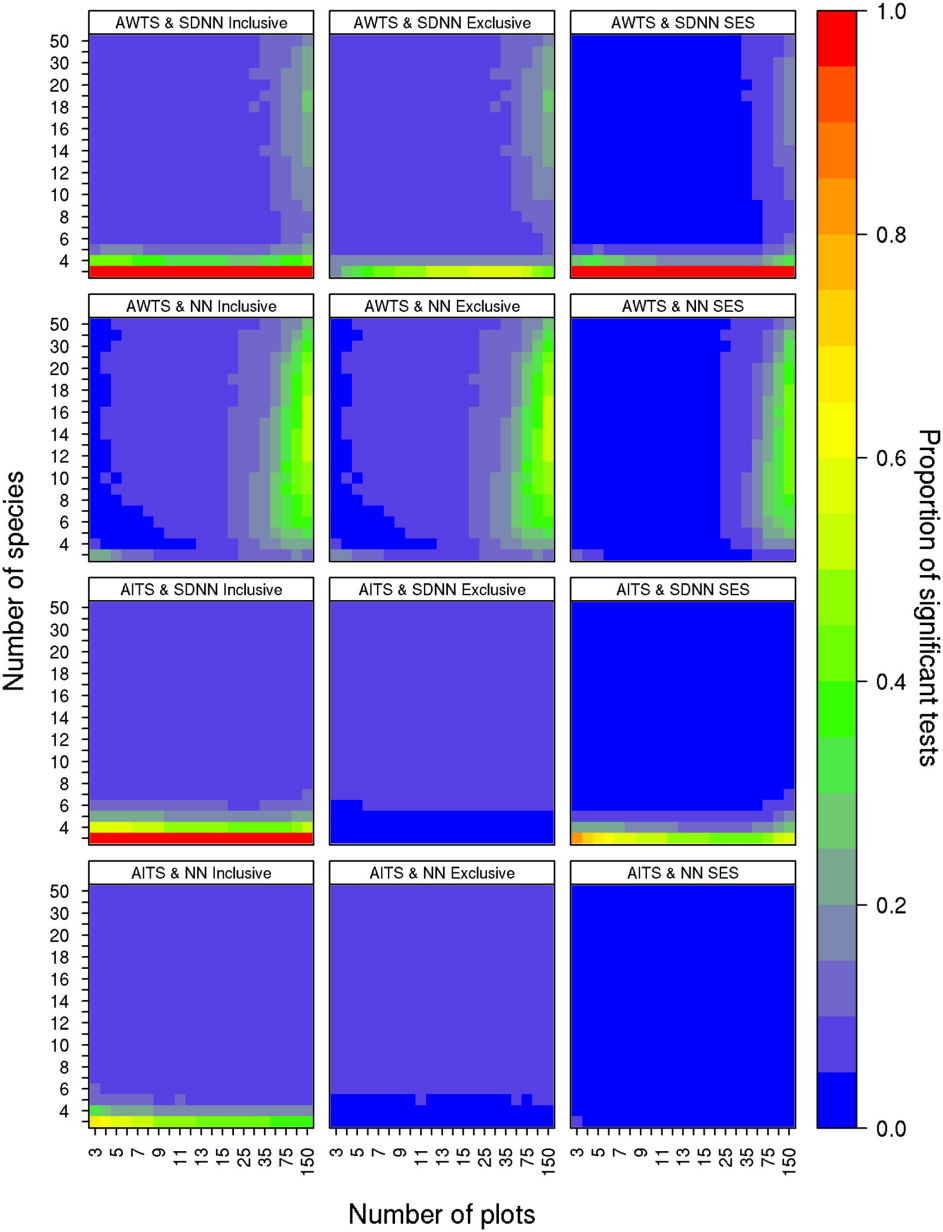
Type I error rates of the limiting similarity null models. Each panel represents a different combination of randomization algorithm, metric and criterion for determining the significance of the null model: inclusive p-values (≤ or ≥), exclusive p-values (< or >), and SES. The colour of each cell indicates the proportion of the 10,000 null models that were significant for that combination of species by sites. Blue cells indicate lower type I error rates and red cells indicate higher type I error rates.

Type I error rates for all three null models were sensitive to both the dimension of the matrices and the method of determining significance (Figs 1 & 2). Co-occurrence null model type I error rates increased with both decreasing species and decreasing site numbers; however, the number of species had greater impact. The use of an inclusive p-value for determining significance resulted in type I error rates between 0.10 and 0.20 (Fig 1). Using exclusive p-values resulted in type I error rates of less than 0.10 (Fig 1) with the majority of tests producing desirable type I error rates (< 0.05). Using SES to determine significance resulted in type I error rates exceeding 0.05 for test matrices containing fewer than five species (Fig 1). For matrices with three species the error rate increased with increasing site number. Using SES for matrices with greater than five species resulted in error rates ≤ 0.05.

For limiting similarity tests, the AITS randomization scheme in combination with the NN metric resulted in type I error rates below 0.05 in all cases when an exclusive p-value was used (Fig 2). Type I error rates were unacceptably high (> 0.05) when inclusive p-values were used in combination with six species or less. The use of SES with three × three matrices also resulted in type I error rates higher than 0.05 (Fig 2). Using AITS in combination with SDNN produced similar results; type I error rates exceeded 0.05 for both the inclusive p-value and SES measures for matrices with less than seven and six species respectively. The AWTS randomization scheme resulted in similar patterns, with the exception that error rates increased above 0.05 when the number of sites in the community matrix surpassed 50 (Fig 2).

### Type II Error Rate Estimation

**Fig 3:**
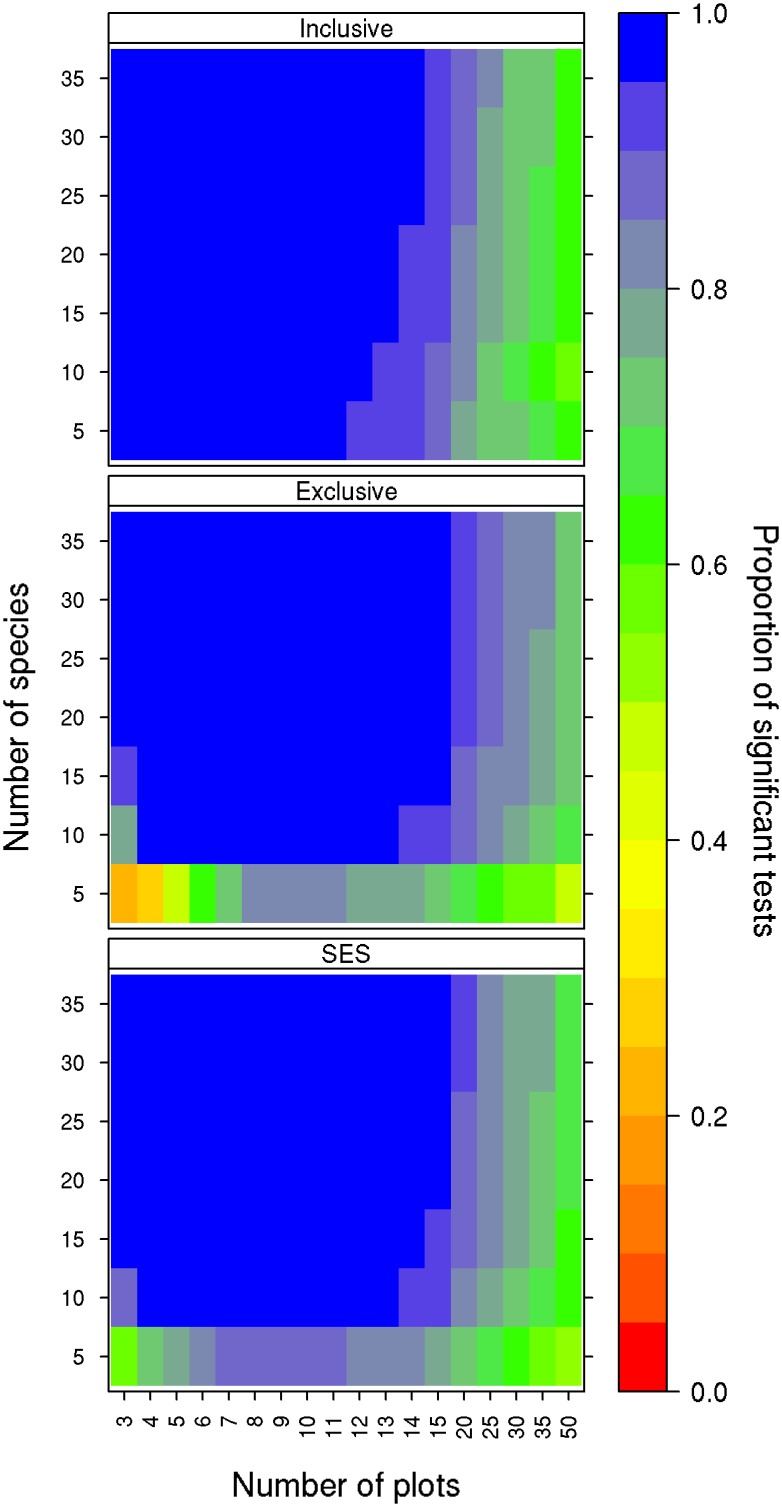
Type II error rates of the co-occurrence null model test. Each panel represents a different criterion for determining the significance of the null model: inclusive p-values (≤ or ≥), exclusive p-values (< or >), and SES. The colour of each cell indicates the proportion of the 10,000 null models that were significant for that combination of species by sites. Blue cells indicate lower type II error rates and red cells indicate higher type II error rates.

When signal is maximized for the focal matrix, negative co-occurrence tests using the inclusive significance criterion detected negative co-occurrence patterns in at least 95% of the test matrices when there were fewer than thirteen sites; however, this rate decreased to 50% as the number of sites increased to 50 (Fig 3). The number of species in matrices had only a marginal effect on type II error rates regardless of the significance criterion used; increasing species number resulted in slightly improved type II error rates for matrices with 12 or more sites (Fig 3).

**Fig 4:**
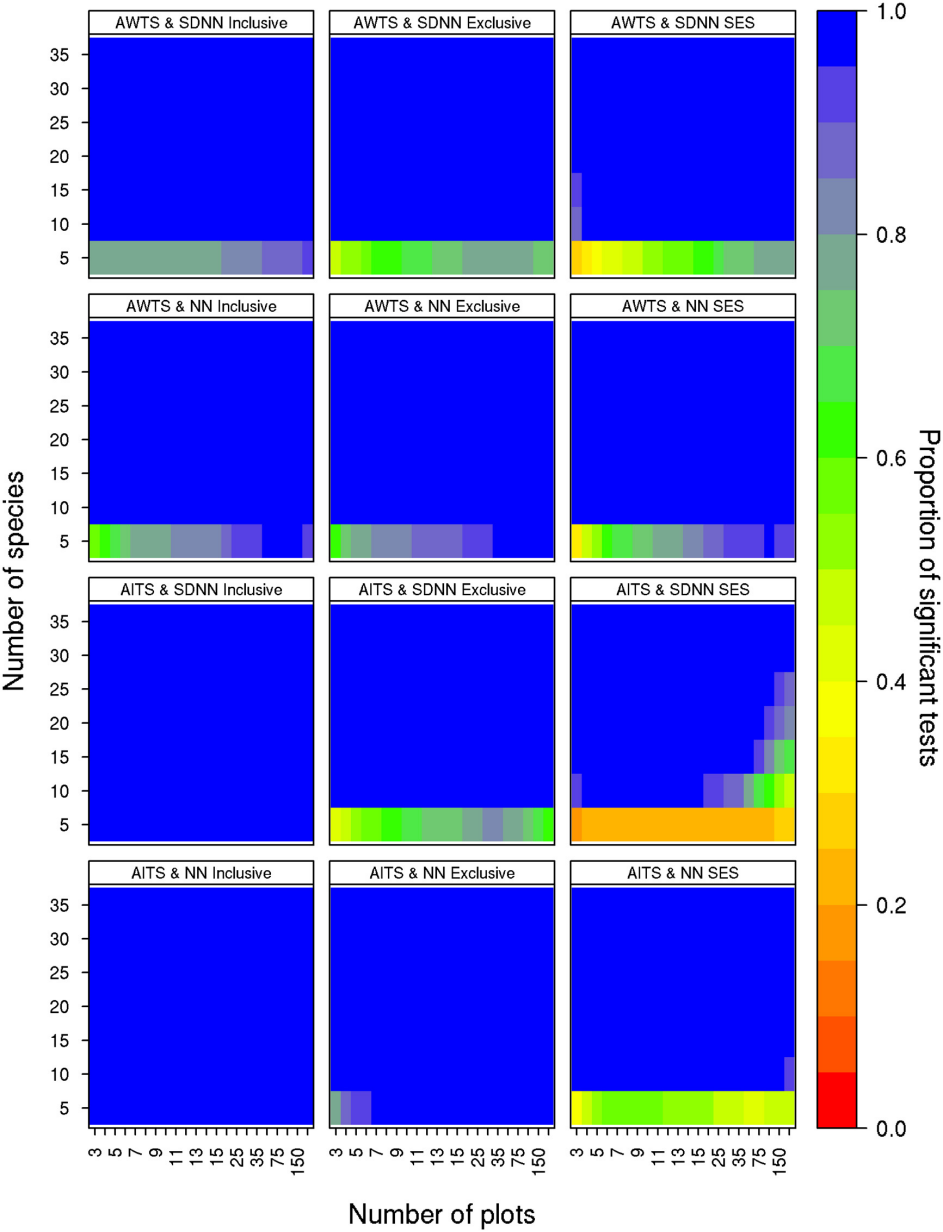
Type II error rates of the limiting similarity null models. Each panel represents a different combination of randomization algorithm, metric and criterion for determining the significance of the null model: inclusive p-values (≤ or ≥), exclusive p-values (< or >), and SES. The colour of each cell indicates the proportion of the 10,000 null models that were significant for that combination of species by sites. Blue cells indicate lower type II error rates and red cells indicate higher type II error rates.

For the limiting similarity null models, type II error rates were generally stable with α ≤ 0.05 and the three significance criteria produced similar results with three exceptions (Fig 4). First, type II error rates did not exceed 0.05 for any of the matrix dimensions tested when an inclusive p-value was used with AITS and either NN or SDNN but did exceed 0.05 for matrices with fewer than 10 species when the exclusive p-value was used (Fig 4). Second, using AITS with SDNN and SES resulted in increasing type II errors as the number of sites increased; however, increasing the number of species counteracted this effect (Fig 4). Third, using the AWTS randomization resulted in similar error rates regardless of metric or significance criteria (Fig 4) with all combinations producing error rates in excess of 0.05 when matrices had fewer than 10 species. Finally, with fewer than 10 species, AWTS error rates decreased as the number of sites increased, but remained above 0.05, (Fig 4).

## Discussion

### Type I Error Rate Estimation

#### Negative co-occurrence

Our results indicate that negative co-occurrence null models become susceptible to type I errors as the number of species decreases, with the rate of decline dependent on the method used to assess significance (Fig 1). Determining significance with an inclusive p-value resulted in unacceptable error rates for matrices with smaller numbers of species (< 8), and also resulted in an overall error rate of approximately 10% for matrices with between eight and 50 species (Fig 1). While the type I error rate of 10% is consistent with the findings of Gotelli [25], it can be improved upon by using an exclusive p-value. The use of an exclusive p-value, which is uncommon for null model tests at this time, resulted in type I error rates below 10% for all matrix dimensions considered with most below five percent (Fig 1).

The use of an inclusive p-value is associated with higher error rates, particularly when examining small matrices, most likely because each matrix can only be shuffled a limited number of ways with a limited number of unique C-Score values. Thus, the likelihood of randomization reproducing the original “observed” matrix (and in our case, C-Score value) increases for smaller matrices; this is clearly undesirable. Any randomization scheme that imposes constraints on how matrices are shuffled is susceptible to this effect with negative impacts more likely with increasingly strict constraints. Solutions include using larger matrices (10 × 10 or larger for example) or using a randomization scheme that is less constrained; however, it is important to balance the constraints on randomization with the goal of appropriately isolating the biological process under investigation against statistical constraints. In larger matrices, however, an exclusive p-value may be less important, as the phenomenon of repeated matrices should diminish quickly with matrix size. It remains unclear why, for larger matrices (> 15 species x sites), type I error rates are improved relative to using the exclusive p-value, although it is possible that the fixed-fixed randomization scheme is sufficiently restrictive that randomization still produces many repeated matrices or equivalent C-Score values, even when the focal matrix is large. Further investigation is warranted.

Our results suggest that the exclusive p-value should be used to assess significance for null model tests to minimize type I error rates. SES provides a convenient way to compare the results between matrices; however, its utility is impacted by the deviance from normality of the null distribution; this may be of particular concern when null distributions among matrices being compared differ in the degree to which each deviates from normality. Alternative effect sizes exist (e.g., [61]) and may serve as a useful alternative.

#### Limiting similarity—AITS

The AITS randomization performed well for both the NN and SDNN metrics when the inclusive p-value was used and the number of species was greater than five and six respectively regardless of the number of sites (Fig 2). However, like the negative co-occurrence null model, error rates for matrices with fewer species (< 6) remained below five percent when an exclusive p-value was used (Fig 2). As with the negative co-occurrence models, this likely results from the generation of random matrices that match the observed matrix being tested. Unlike the co-occurrence null model using an exclusive p-value does not result in improved error rates with larger matrices. This is likely due to the way the metrics are calculated and the fact that they are less granular than the C-Score metric. Using SES improved the type I error rates for the AITS-NN combination compared to the inclusive p-value as well as for the AITS-SDNN, albeit moderately (Fig 2).

#### Limiting similarity—AWTS

Limiting similarity tests using AWTS and NN had excellent type I error rates for matrices with as few as four species; there was, however, a significant increase in type I error rates with increasing site number (Fig 2). This trend was consistent for all three significance criteria with error rates exceeding 10% for matrices with more than 35 sites (Fig 2).

For AWTS in combination with SDNN, type I error rates for matrices with greater than five species were very good (< 5%); however, error rates increased with the number of sites, exceeding 10% for matrices with 35 or more sites (Fig 2). It is not clear why this increase occurs.

We performed post-hoc analyses to determine the source of increased type I error rates with the AWTS null model. We compared trait abundances pre- and post-shuffle using a Chi-Square goodness of fit test and found that patterns of trait abundance, which AWTS should maintain, are more poorly maintained as matrix size increases (S2 Fig). We also examined the variance (breadth) of the null model distributions in relation to matrix size (increasing site and/or species number) because a narrowing of the null distribution can lead to increased type I errors. The standard deviation of the null distributions decreased with both increasing site number and increasing species number (S3 Fig). The narrowing of the null distribution with increasing site number appears to result from a combination of the underlying log-normal abundance distribution and the AWTS algorithm itself. As the number of sites increases, the proportion of species with abundant traits increases logarithmically. The effect of this is twofold: first, the contribution of the abundant traits quickly overwhelms any contribution of less abundant traits to the overall community metric (NN or SDNN); second, in the AWTS algorithm, the way traits are shuffled becomes more constrained with increasing matrix size. As the number of sites increases, differences in abundance among the most common species become so extreme that the AWTS results in little variation in abundant trait values among sites, which results in narrow null distributions. It is not clear if this is a problem for studies that have used this kind of randomization, as implementations of AWTS likely vary in small but potentially significant ways; however, this is likely to be an issue with this combination of null model (AWTS) and metrics (NN and SDNN) particularly when the number of sites and/or species is high and species occurrences are log-normally distributed (e.g., [17]).

### Type II Error Rate Estimation

#### Negative co-occurrence

Our results indicate that type II error rates for the negative co-occurrence null model are acceptable for matrices with fewer than 15 sites but increased with increasing site number beyond this; variation in species number had little impact (Fig 3). We suspected that the observed increase in type II errors with increasing site number may have resulted from the method used to add structure to matrices; our method searched 10,000 matrices to maximize C-Score, but this is a shrinking fraction of possible matrices as matrix size increases. To investigate, we ran a reduced set of null model tests for a set of matrices with 35 sites and increased the number of shuffles used to maximize the C-Score from 10,000 to 1,000,000. This post-hoc analysis supported our suspicion; increasing the number of matrices considered in maximizing C-Score clearly reduced the type II error rate in larger matrices (S4 Fig). As such, co-occurrence tests appear to have acceptable type II error rates in general across a wide range of matrix sizes, when signal is maximized for the test matrix. It is clear, however, that error rates for small matrices are generally the result of including identical matrices in the null distribution. Using the exclusive p-value for these tests is advisable, although care should be taken in using very small matrices that may not adequately represent the species co-occurrence patterns that exist within the study system.

#### Limiting similarity-AITS

AITS in combination with the NN or SDNN test statistic had good type II error rates (< 0.05) for matrices with more than 10 species (Fig 4). While statistical power for combinations of AITS and the test statistics was good in general, it was best when the inclusive p-value, very good when the exclusive p-value was used, and worst when SES was used in combination with lower species numbers and higher site numbers (Fig 4). It is advisable to use the exclusive p-value criterion, as it results in low type I error rates and still has relatively strong statistical power. The exclusive p-value also reduces the potential for significant results deriving from null distributions built from repeat matrices. For example, traits may only be shuffled amongst five species 120 times meaning a null distribution of 5000 will inappropriately contain many repeats.

#### Limiting similarity-AWTS

AWTS in combination with either test statistic had good type II error rates for most matrix sizes, although these error rates were generally greater than 10% when matrices contained fewer than ten species (Fig 4). Type II error rates were consistent for AWTS regardless of the significance criterion used (Fig 4). For this null model, error rates for small matrices cannot be ameliorated through the use of the exclusive p-value, as the null model performs poorly for that size class in general (Fig 4). However, as the exclusive p-value is more conservative in assessing significance and does not appear to negatively impact this null model test, it seems advisable to use an exclusive p-value for all tests using the AWTS randomization. With the exception of matrices containing fewer than five samples or sites, this model demonstrated good statistical power across a wide spectrum of matrix sizes, consistent with the findings of Hardy [26] for an analog of this randomization procedure as used to assess phylogenetic dispersion.

## Conclusion and Recommendations

Growing use of null models by ecologists makes it imperative that we understand the statistical properties of these models and whether they are stable across matrix sizes. We evaluated error rates for two common classes of null model that are used to assess either negative co-occurrence or trait-based limiting similarity. Type II error rates were examined for matrices in which the signal for the expected patterns was maximized.

All null models generally performed better when the exclusive p-value was used. In general, the nature of null models is that they will vary in the degree to which the randomization scheme will produce identical matrices or metric values. Using the exclusive p-value is a more conservative approach to assessing significance and is prudent to use in combination with small focal matrices, as this conservatism doesn’t appear to strongly impact type II error rates.

Our results suggest a minimum safe standard matrix size; the threshold varies with null model, but safe practice would be to use these models with matrices containing ≥10 species; below this, error rates increased unacceptably. For limiting similarity models using the AWTS algorithm, type I error rates became undesirably high when matrices with more than 35 sites where tested. Limiting similarity null models using the AITS algorithm had good error rates in general for both metrics. An important qualification of these results is that these error rates are good when the signal for the pattern of interest comes from 100% of species in the focal matrix. It remains to be seen whether error rates are acceptable when signal comes from fewer species.

## Supporting Information

S1 Fig Standardized effect size (SES) of the limiting similarity null models in relation to the SES of the co-occurrence null model for the same matrix. All C-Score SES values are positively skewed. The SES values of the limiting similarity null models shown in panels b, c and d indicate that there is some interaction between C-Score SES and limiting similarity SES values (SDNN & AITS: r = -0.0011, p = 0.0245; mean NTD & AITS: r = -0.0001, p = 0.7572; mean NTD & AWTS: r = 0.0002, p = 0.6062; SDNN & AWTS: r = -0.0002, p = 0.631).
(TIFF)

S2 Fig Chi-Square values for goodness of fit test comparing trait abundance before and after shuffling matrices of different sizes. The top panel shows the results for the abundance weighted trait shuffling algorithm (AWTS). The bottom panel are the results for the abundant independent trait shuffling algorithm (AITS). AWTS fails to maintain trait abundances when matrices have more than 200 sites. Trait abundances with AITS, which is not intended to maintain trait abundance, leads to significant differences in trait-abundance relationships with very small matrices. The horizontal dashed line represents the critical χ^2^ value (χ^2^ = 30.1435, df = 19, α = 0.05).
(TIFF)

S3 Fig Type II error rates of the co-occurrence null model test with respect to the number of shuffles used to introduce structure into the matrices. Each panel represents a different criterion for determining the significance of the null model. The colour of each cell indicates the proportion of the 10,000 null models that were significant for that combination of species by sites. Blue cells indicate lower type II error rates and red cells indicate higher type II error rates. 10k = 10,000 and 1M = 1,000,000
(TIFF)

S4 Fig Mean standard deviations of the null distribution for each species × site combination. Each cell in the plot (species × site) represents average standard deviation of 10,000 null distributions. Increasing plot number (and number of species) results in narrower (lower mean standard deviation) null distributions. This narrowing of the null distribution contributes to the increased rate of type I errors with increasing plot number.
(TIFF)
