# Supplementary figures and images for: The Influence of Matrix Size on Statistical Properties of Co-Occurrence and Limiting Similarity Null Models
Source: PLoS One. 2016 Mar 4;11(3):e0151146. doi: 10.1371/journal.pone.0151146 (PMC4778770; doi:10.1371/journal.pone.0151146)

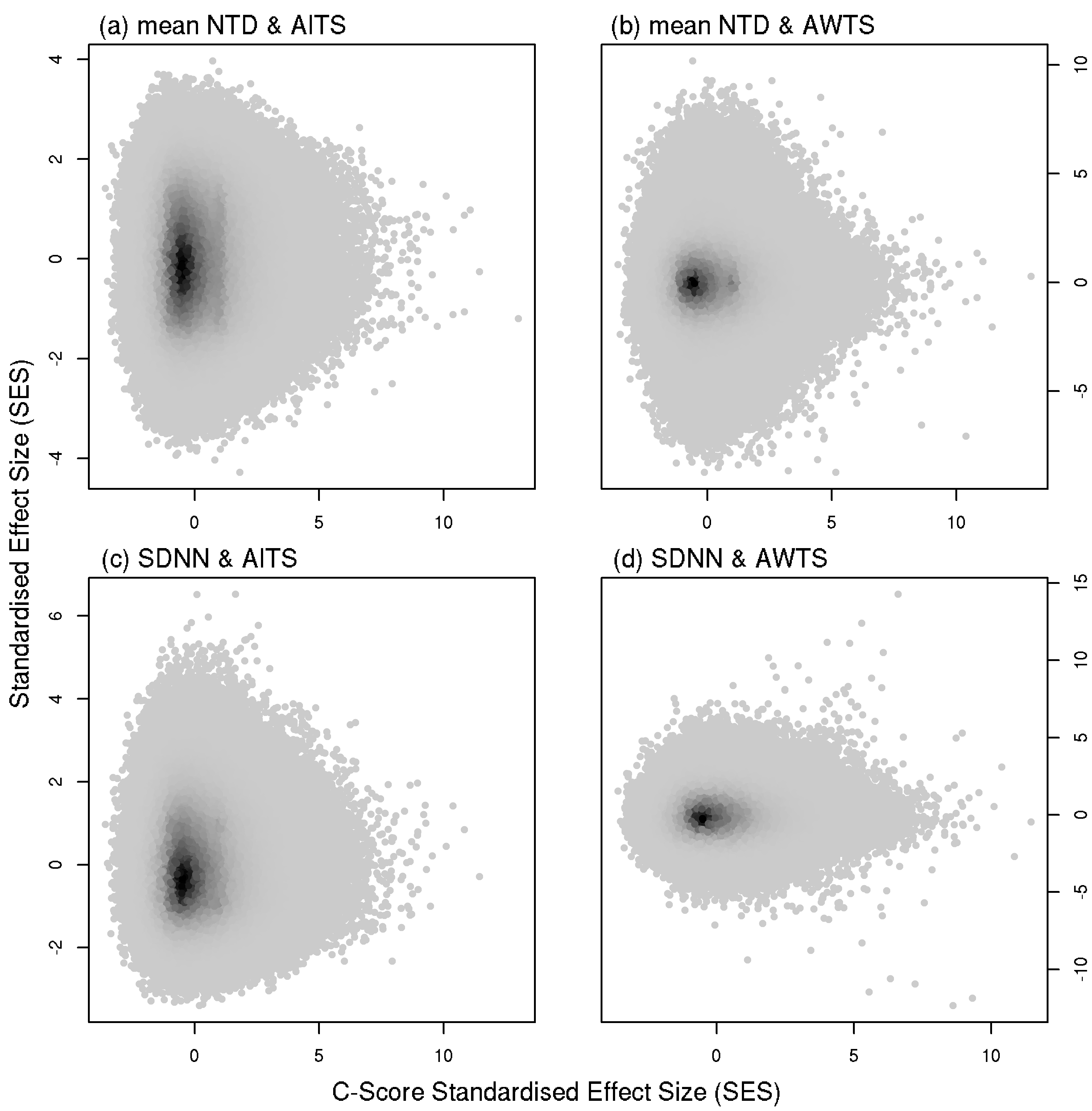

Supplement: S1 Fig — All C-Score SES values are positively skewed. The SES values of the limiting similarity null models shown in panels b, c and d indicate that there is some interaction between C-Score SES and limiting similarity SES values (SDNN & AITS: r = -0.0011, p = 0.0245; mean NTD & AITS: r = -0.0001, p = 0.7572; mean NTD & AWTS: r = 0.0002, p = 0.6062; SDNN & AWTS: r = -0.0002, p = 0.631). (TIFF) [file pone.0151146.s001.tiff]

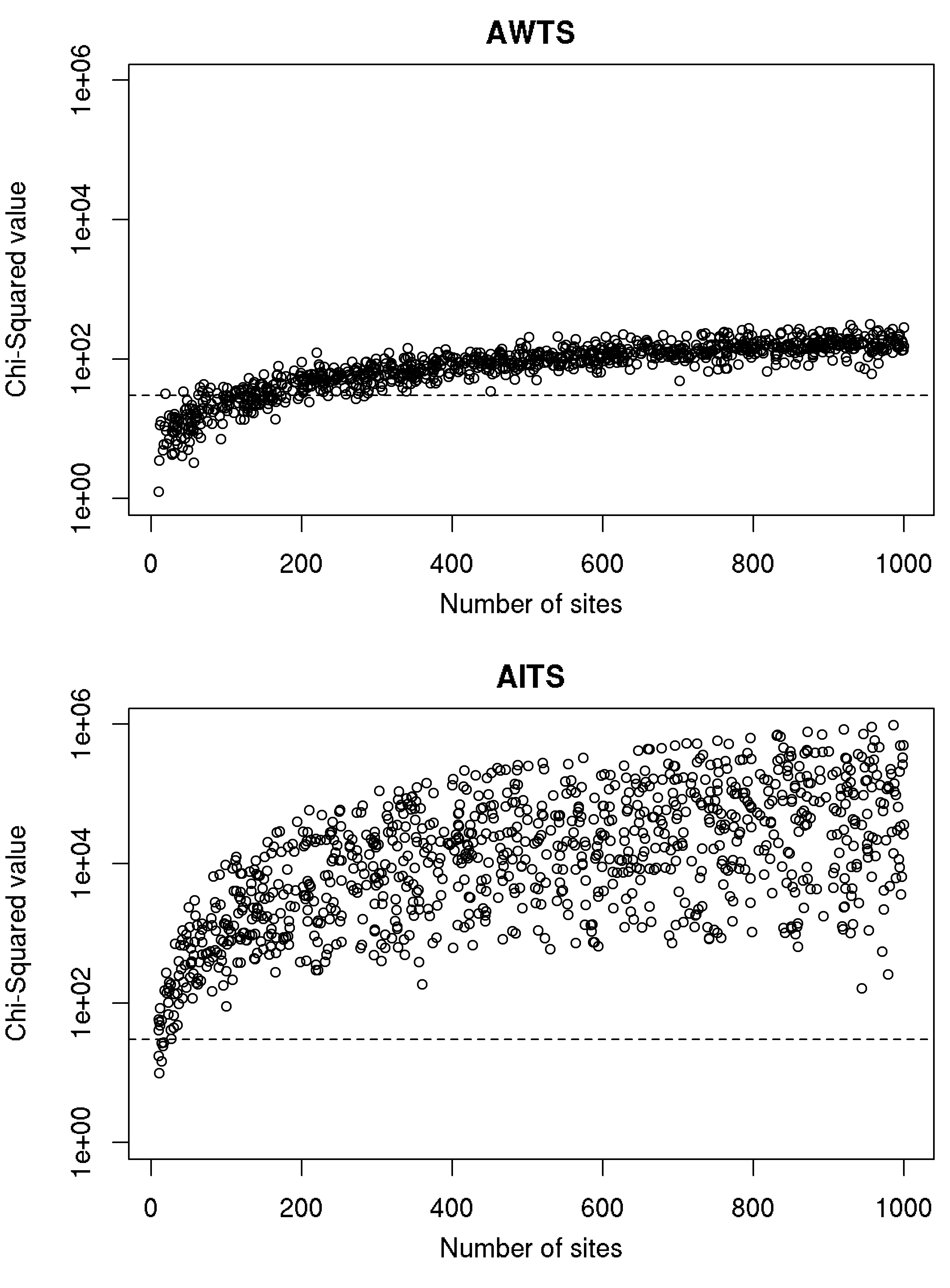

Supplement: S2 Fig — The top panel shows the results for the abundance weighted trait shuffling algorithm (AWTS). The bottom panel are the results for the abundant independent trait shuffling algorithm (AITS). AWTS fails to maintain trait abundances when matrices have more than 200 sites. Trait abundances with AITS, which is not intended to maintain trait abundance, leads to significant differences in trait-abundance relationships with very small matrices. The horizontal dashed line represents the critical χ2 value (χ2 = 30.1435, df = 19, α = 0.05). (TIFF) [file pone.0151146.s002.tiff]

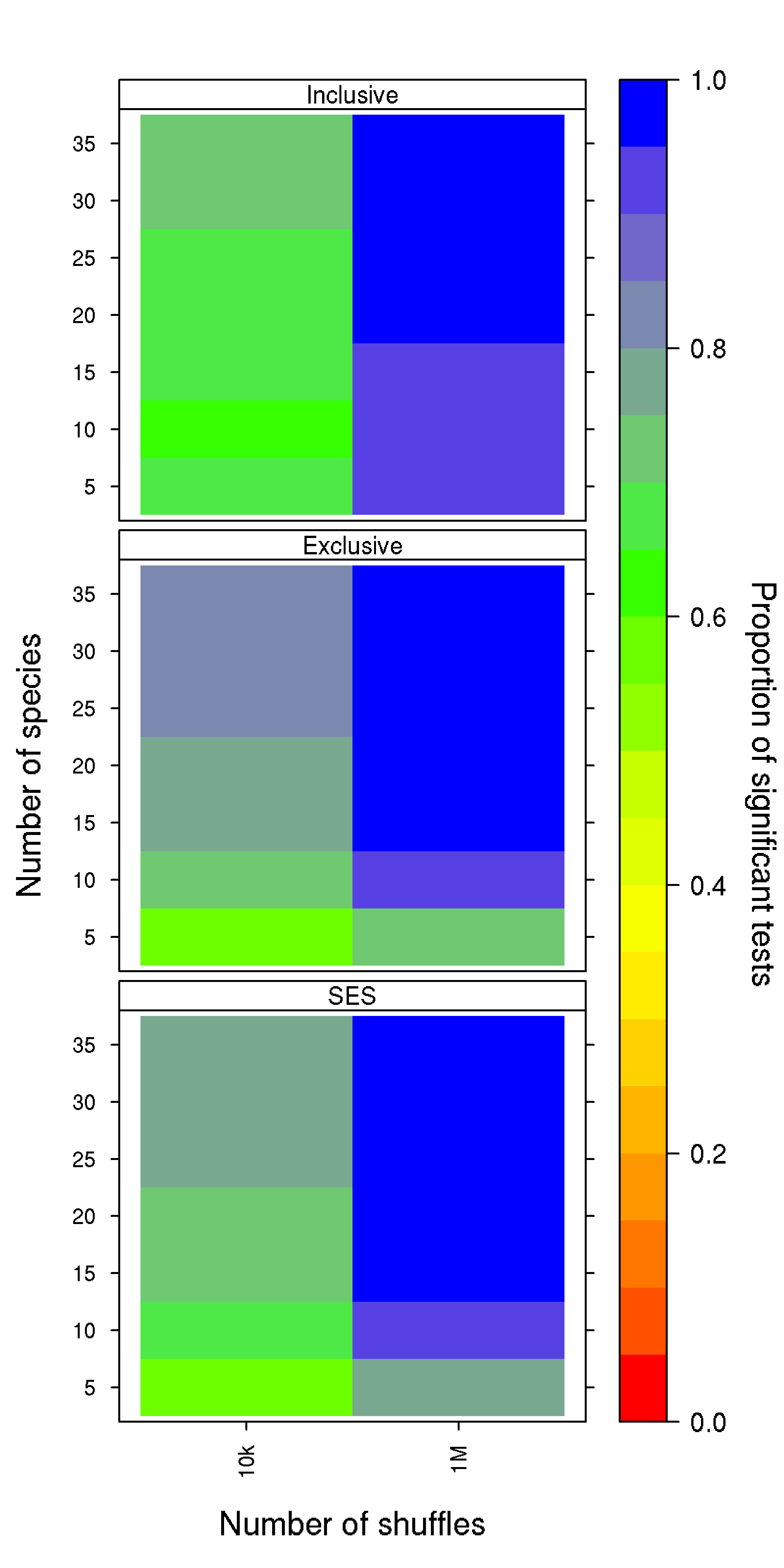

Supplement: S3 Fig — Each panel represents a different criterion for determining the significance of the null model. The colour of each cell indicates the proportion of the 10,000 null models that were significant for that combination of species by sites. Blue cells indicate lower type II error rates and red cells indicate higher type II error rates. 10k = 10,000 and 1M = 1,000,000 (TIFF) [file pone.0151146.s003.tiff]

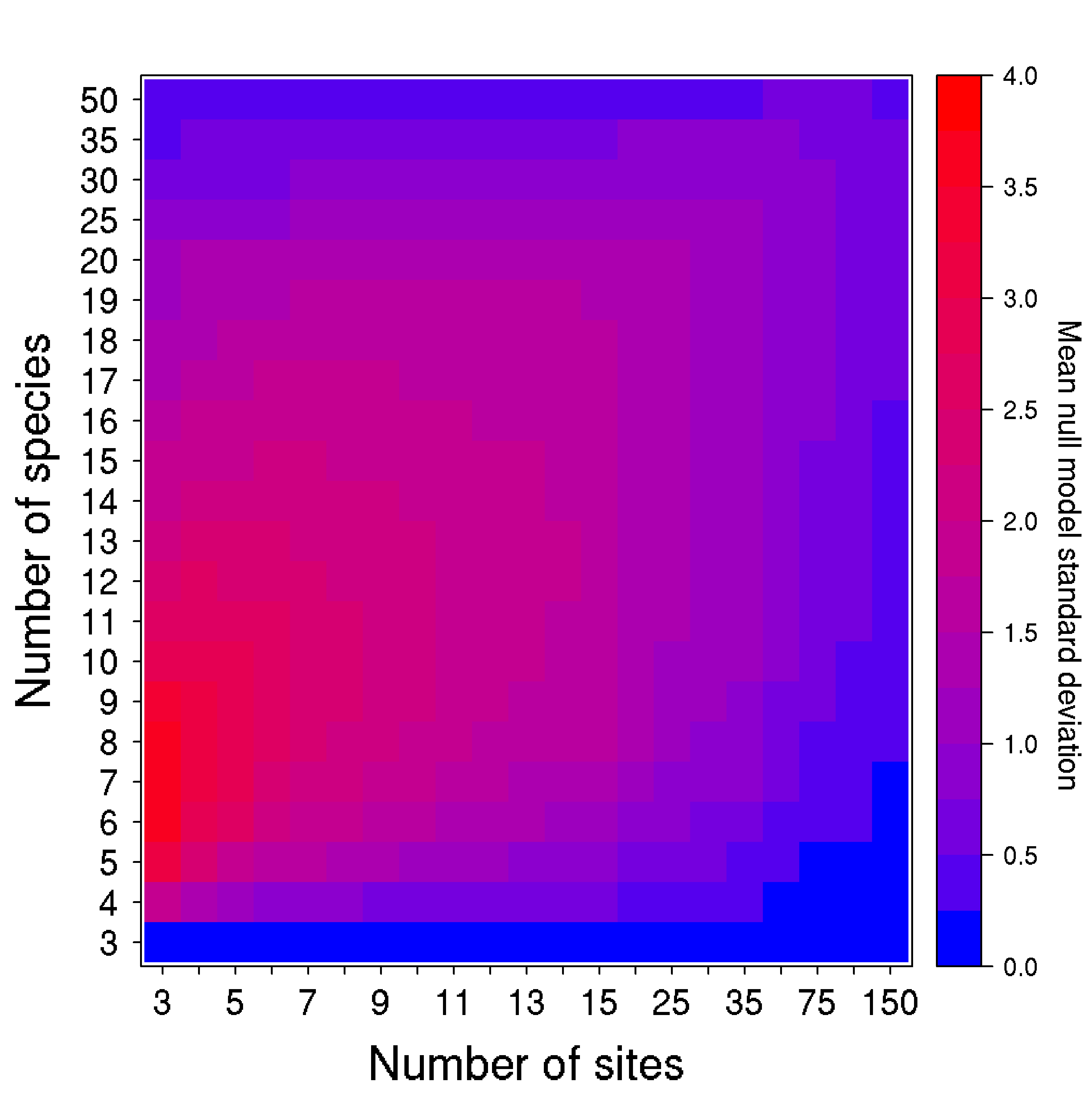

Supplement: S4 Fig — Each cell in the plot (species × site) represents average standard deviation of 10,000 null distributions. Increasing plot number (and number of species) results in narrower (lower mean standard deviation) null distributions. This narrowing of the null distribution contributes to the increased rate of type I errors with increasing plot number. (TIFF) [file pone.0151146.s004.tiff]
